# Polycyclic Tetramate Macrolactams—A Group of Natural Bioactive Metallophores

**DOI:** 10.3389/fchem.2021.772858

**Published:** 2021-11-12

**Authors:** Ling Ding, Sheng-Da Zhang, Ahmad Kasem Haidar, Manila Bajimaya, Yaojie Guo, Thomas Ostenfeld Larsen, Lone Gram

**Affiliations:** Department of Biotechnology and Biomedicine, Technical University of Denmark, Kongens Lyngby, Denmark

**Keywords:** tetramate, Fenton chemistry, metallophore, PTM, antibiotics

## Abstract

New infectious diseases and increase in drug-resistant microbial pathogens emphasize the need for antibiotics with novel mode-of-action. Tetramates represented by fungi-derived tenuazonic acid and bacterial polycyclic tetramate macrolactams (PTMs) are an important family of natural products with a broad spectrum of antimicrobial activities. Despite their potential application as new antibiotics, it remains unknown how PTMs function. In this study, genomic mining revealed that PTM biosynthetic gene clusters (BGCs) are widespread in both Gram-positive and Gram-negative bacteria, and we investigated a sponge endosymbiont *Actinoalloteichus hymeniacidonis* harboring a potential PTM-BGC. Xanthobaccin A that previously has only been isolated from a Gram-negative bacterium was obtained after a scale-up fermentation, isolation, and structure elucidation through mass spectrometry and nuclear magnetic resonance (NMR) spectroscopy. Xanthobaccin A as well as two previously reported tetramates, equisetin and ikarugamycin, exhibited antibacterial activities against *Bacillus subtilis*. In addition, these three tetramates were for the first time to be confirmed as metallophores and the stoichiometry of the complexes were shown to be Fe(III)(equisetin)_3_/Fe(III)(equisetin)_2_ and Fe(III)(ikarugamycin)_2_, respectively. Meanwhile, we found that all three tetramates could reduce ferric into ferrous iron, which triggers the Fenton chemistry reaction. Their antibacterial activity was reduced by adding the radical scavenger, vitamin C. Altogether, our work demonstrates that equisetin and PTMs can act as metallophores and their antimicrobial mechanism is possibly mediated through Fenton chemistry.

## Introduction

The increase in drug-resistant pathogenic microorganisms is a major societal challenge (Cooper and Shlaes, 2011) and the development of antibiotics with novel mode-of-action is urgently needed. Natural products represent an important source of drugs, and more than 50% of approved new antibiotics are either natural products or natural products-derived (Newman and Cragg, 2020). Therefore, one promising drug discovery strategy is to further explore microbial natural products.

**FIGURE 1:**
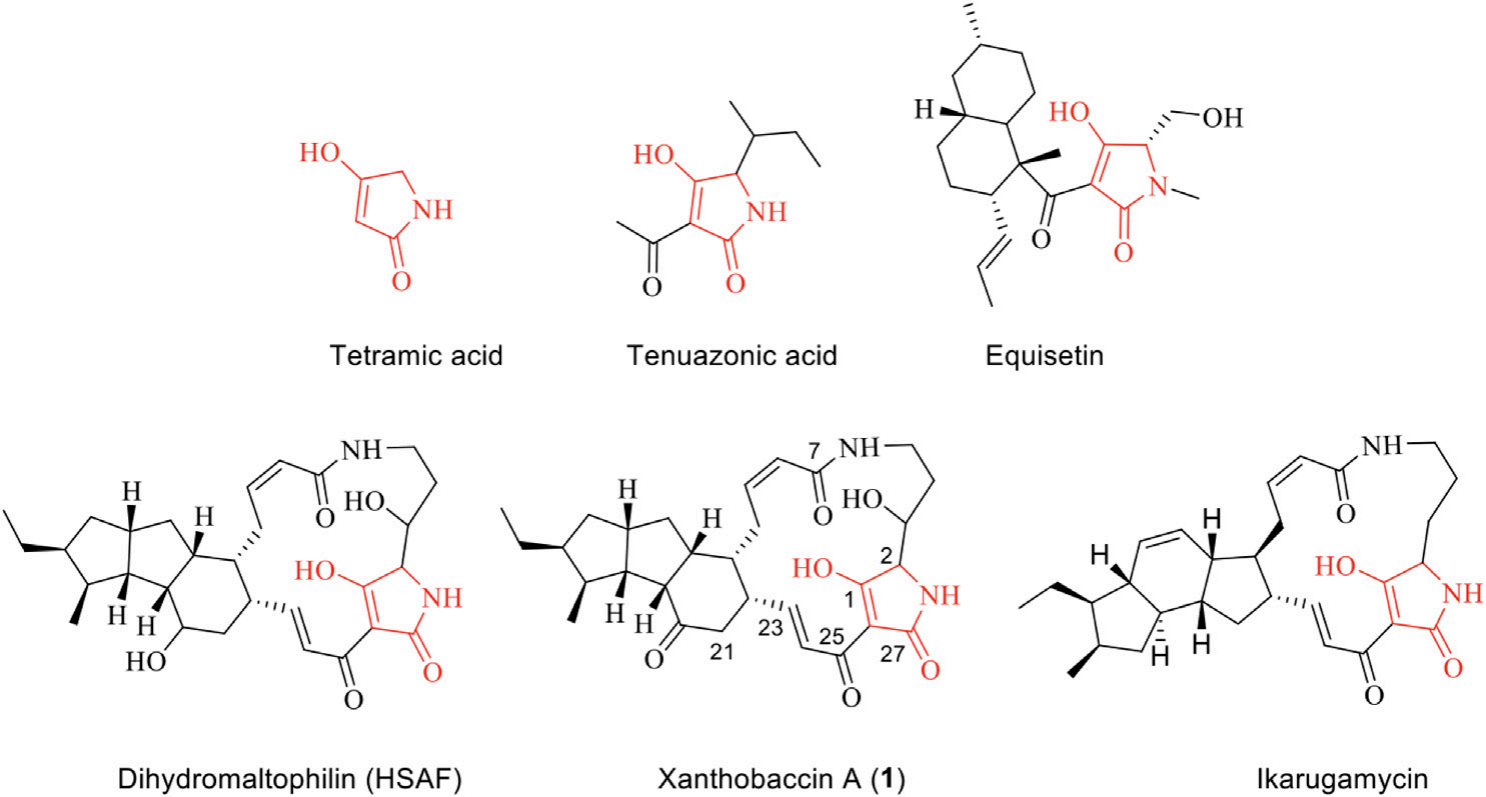
Chemical structure of tetramic acid and selected examples of natural products containing a tetramic acid unit.

Natural products with metal-chelating properties have a great potential for the development of new antibiotics. Polyphenols, quinones, 3-acyltetramic, and tetronic acids are among those natural products with metal-chelating properties, and some derivatives exhibit profound activities against multidrug-resistant bacteria (Dandawate et al., 2019). For example, natural products containing a tetramate-moiety (Figure 1) represent an important class of bioactive compounds with a broad spectrum of antimicrobial activities. There are two well-known examples, namely the fungal natural products equisetin (Vesonder et al., 1979) and tenuazonic acid (Stickings 1959). Tenuazonic acid is a toxic constituent from *Alternaria tenuis* Auct, *Phoma sorghina*, and other phytopathogenic fungi (Laatsch 2012). Tenuazonic acid can complex with copper, iron, nickel, and magnesium ions (Lebrun et al., 1985) and it has been suggested that the biological activity of tetramates is related to their metal-complexing ability (Steyn and Rabie 1976). The crystal structure of copper bis (tenuazonate) monohydrate has been determined by X-ray crystallography (Dippenaar et al., 1977). Although enolic tautomers of tenuazonic acids exist, their crystal structure has revealed a square-planar Cu(II) complex with a Z-enol form in which the amide and acetyl oxygen atoms are bound to the metal (Dippenaar et al., 1977). The complexation of tenuazonic acid with iron(III), nickel(II), and magnesium(II) was further investigated in 1985. Mass spectroscopy and IR spectra provided evidence of stoichiometry of Fe (III)(TA)_3_, Ni(II)(TA)_2_, and Mg(III)(TA)_2_ (Lebrun et al., 1985). The addition of FeCl_3_ and MgCl_2_ did not reverse the toxicity to bacteria or rice cells indicating that the activity is not due to deprivation of these essential metals (Lebrun et al., 1985). Herein the mode-of-action remains unknown.

In comparison to the fungal tetramates, bacteria tend to produce polycyclic tetramate macrolactams (PTM), which are an emerging class of natural products, that includes the antifungal HSAF produced by a Proteobacterium (Yu et al., 2007), the antinematode geodin A from a sponge (Capon et al., 1999), the antiprotozoal ikarugamycin from a *Streptomyces* sp. (Jomon et al., 2012), and the anticancer compounds ikarugamycins and clifednamide A from a sponge-associated *Streptomyces* sp. (Dhaneesha et al., 2019). Intriguingly, many bacterial PTM producers are involved in a beneficial association with higher organisms. Due to their biological activities, several of these microorganisms have been developed as biocontrol agents in agriculture, e.g. the HSAF-producer *Lysobacter enzymogenes* (Yu et al., 2007) and *Stenotrophomonas* sp., the latter of which lives in the sugar beet rhizosphere and produces the antifungal agent xanthobaccin A active against the host-pathogen *Pythium ultimum* (Hashidoko et al., 1999). Despite their important biological activities, the mode of action of PTMs and other tetramates remains elusive. PTMs harboring a tetramate moiety could potentially act as metal chelators, however, this has, to our knowledge, not been investigated. Hence, we aim to provide new evidence on how the larger tetramates complex with ions and how tetramates broadly function.

**FIGURE 2:**
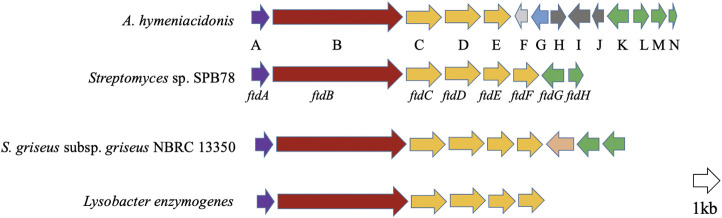
Open reading frame (ORF) map of the possible xanthobaccin biosynthetic locus from *A. hymeniacidonis* compared to other characterized PTM clusters in *Streptomyces* sp. SPB78 (frontalamides, Blodgett et al., 2010), *S. griseus* subsp. *griseus* NBRC 13350 (frontalamide-like compounds, Luo et al., 2013) and *Lysobacter enzymogenes* (HSAF, Yu et al., 2007). Each ORF is color-coded to designate ftd orthologs. Those shown in purple encode for sterol desturase, red for hybrid PKS-NRPS, orange for oxidoreductase and green for transporters. For *A. hymeniacidonis* (Supplementary Table S1), there are siderophore interaction-related proteins F (siderophore-interacting protein) and G (iron-siderophore-interacting ABC transporter substrate-binding protein); H–J: proteins with unknown functions.

The starting point for the investigations was a genome-mining survey on numerous bacterial genomes, which revealed that PTMs are widespread in both Gram-positive and Gram-negative bacteria (Supplementary Figure S1). Among those bacteria, *Actinoalloteichus hymeniacidonis*, an endosymbiont from the sponge *Hymeniacidon perlevis* (Zhang et al., 2006) was found to harbor a potential PTM biosynthetic gene cluster (Figure 2). Analysis of the gene cluster showed the presence of putative genes coding for siderophore interacting proteins downstream of the key PKS-NRPS gene. This indicated that the product could be a metallophore. We, therefore cultivated the bacterium under iron-deficient conditions which led to the production of putative PTMs. From the 6 L fermentation broth, we isolated and characterized the antimicrobial component as xanthobaccin A. Further analysis revealed that xanthobaccin A, together with two other microbial tetramates, ikarugamycin and equisetin, can chelate ferric iron and reduce it to ferrous iron triggering the cascade of Fenton chemistry. In this paper, we describe the antimicrobial, iron-chelating, and antimicrobial mechanisms of xanthobaccin A together with equisetin and ikarugamycin.

## Materials and Methods

### Bacterial Strains and Culture Conditions

*Actinoalloteichus hymeniacidonis* HPA 177T was purchased from DSMZ, Germany. *Bacillus subtilis* ATCC6051 was obtained from the research group of Professor Ákos T. Kovács, Department of Biotechnology and Biomedicine, Technical University of Denmark.

### Genome Mining

To identify, annotate and analyze the secondary metabolite biosynthetic gene clusters, NCBI BLAST (Boratyn et al., 2013) and AntiSMASH 5.0 (Blin et al., 2019) were used in the genome mining process of *A. hymeniacidonis* (CP014859.1) and other PTM producers (Supplementary Table S2).

### Accession Numbers of Source Files Used to Compare Unknown *ftd*-Like Gene Clusters in Bacterial Genomes

The following GenBank releases are sources of *ftd*-like gene clusters used in comparative analyses: *Streptomyces* sp. SPB78, GenBank accession NZ_ACEU01000453; *S. griseus* subsp. *griseus* NBRC 13350, GenBank access AP009493.1; *Lysobacter enzymogenes*, GenBank access EF028635.2.

### General Chemical Experimental Procedures

NMR spectra were recorded on a Bruker Advance III 800 MHz spectrometer. Silica gel chromatography was performed on silica gel 60 (Merck, 0.04–0.063 mm, 230–400 mesh ASTM) and Sephadex LH20 (Pharmacia). Ikarugamycin and equisetin were ordered from Sigma-Aldrich. Solvents of analytical grades were ordered from VWR.

### Fermentation and Isolation

To obtain sufficient amounts of metabolites for chemical characterization, *A. hymeniacidonis* was cultured in a 6 L liquid fermentation at 28°C, 200 rpm for 7 days. Slant of spores of *A. hymeniacidonis* was inoculated in a 500 ml flask containing 150 ml medium soluble starch 4 g/L, KNO_3_ 2 g/L, NaCl 1 g/L, MgSO_4_·7H_2_O 0.5 g/L, CaCO_3_ 0.02 g/L, yeast extract 1 g/L. The culture broth was separated into supernatant and mycelia parts, respectively. Both were extracted by ethyl acetate and the organic phases were combined. Evaporation of the organic solvent yielded 1.8 g of crude extract that was partially purified in flash chromatography by silica gel chromatography using gradient solutions of dichloromethane and methanol into 10 fractions F1-10. The F7 fraction containing PTMs was further purified by semi-preparative HPLC using an XBridge RP_18_ HPLC Column 10 × 250 mm, 5 μm, a flow rate of 4 ml/min, 40.0°C. Using a 28 min multi-step method and acetonitrile and water as mobile phases the following method was applied in semi-preparative HPLC: at 0–5 min 10–50% acetonitrile, at 5–7 min 50–60% acetonitrile, at 7–15 min 60–80% acetonitrile, at 15–18 min 80–100% acetonitrile, and acetonitrile was maintained at 100% for another 5 min and followed by re-equilibration to 10% acetonitrile until 28 min. Pure compound **1** (0.8 mg) was obtained and analyzed by NMR spectroscopy.

### HPLC-MS Analysis

A UHPLC–DAD–QTOF method was set up for the screening, with an injection volume of 1 μl extract. The separation was performed on a Dionex Ultimate 3000 UHPLC system (Thermo Scientific, Dionex, Sunnyvale, CA, United States) equipped with a 100 × 2.1 mm, 2.6 μm, Kinetex C_18_ column, held at a temperature of 40°C, and using a linear gradient system composed of A: water, and B: acetonitrile. The flow rate was 0.4 ml min^−1^.

Time-of-flight detection was performed using a maXis 3G QTOF orthogonal mass spectrometer (Bruker Daltonics, Bremen, Germany) operated at a resolving power of ∼50,000 full width at half maximum FWHM. The instrument was equipped with an orthogonal electrospray ionization source, and mass spectra were recorded in the range m/z 100–1,000 as centroid spectra, with five scans per second. For calibration, 1 μl of 10 mmol sodium formate was injected at the beginning of each chromatographic run, using the divert valve 0.3–0.4 min. Data files were calibrated post-run on the average spectrum from this time segment, using the Bruker HPC high-precision calibration algorithm.

For ESI^+^ the capillary voltage was maintained at 4,200 V, in the spray chamber, the gas flow to the nebulizer was set to 2.4 bar, the drying temperature was 220°C, and the drying gas flow was 12.0 L min^−1^. Transfer optics ion-funnel energies, quadrupole energy were tuned on HT-2 toxin to minimize fragmentation. For ESI^−^ the settings were the same, except that the capillary voltage was maintained at −2,500 V. Ion-cooler settings were: transfer time 50 µs, radiofrequency RF 55 V peak-to-peak Vpp, and pre-pulse storage time 5 µs.

### Iron-Reducing Assay

The iron II detecting agent ferrozine was used to test the iron-reducing activity of three tetramates, xanthobaccin A, equisetin, and ikarugamycin. A reaction solution comprised of 10 µl test tetramate (1 mg/ml), 10 µl ammonium iron III citrate C_6_H_8_FeNO_7_ (5 mg/ml), and 20 µl aqueous ferrozine (1% wt/vol). FeSO_4_ mixed with aqueous ferrozine (1% wt/vol) was used as a positive control. Tetramate mixed with ammonium iron III citrate was used as a negative control. All components were dissolved in ammonium chloride buffer 1 M, pH 4.5. After 5 min reaction under darkness, the reaction mixtures were analyzed by HPLC-HRMS.

### Antimicrobial Assay

An agar diffusion assay was carried out to test the antimicrobial activity of tetramates against *Bacillus subtilis* ATCC 6051. Whatman Antibiotic assay discs of 6 mm were loaded with 20 µg pure tetramates with/without vitamin C (10 µg). The growth medium for *B. subtilis* was 3 g meat extract, 5 g (Bacto)-peptone, 5 g glucose, 1 L tap water, pH 7.3–7.5, 18 g agar. The test plates were prepared by pouring 14 ml of L-agar as a base layer; after solidifying, this was overlaid with 4 ml of the inoculated seed layer. Roximycin was used as a positive antibiotic control. Pure methanol and vitamin C were used as negative controls. The plates were incubated at 37°C for 24 h, and antimicrobial activity was recorded as clear zones (in mm) of inhibition surrounding the disk. The test sample was considered active when the zone of inhibition was greater than 6 mm. The MIC assay was done by the broth dilution method according to the NCCLS (National Committee for Clinical Laboratory Standards, 1997).

## Results

### Sponge Bacterial Endosymbiont Harbors a Tetramate Biosynthetic Gene Cluster

*Actinoalloteichus hymeniacidonis* HPA 177^T^ is a Gram-positive, rare actinomycete isolated from the marine sponge *Hymeniacidon perlevis* (Zhang et al., 2006). Our genome mining revealed that it harbored a putative PTM BGC (Figure 2), and the core biosynthetic PKS-NRPS protein, which exhibited 67 and 61% similarity to HSAF and frontalamide synthetase proteins, respectively. The individual genes from the PTM gene cluster are listed in Supplementary Table S1 and the proposed biosynthetic pathway is shown in Supplementary Figure S2. The biosynthesis was proposed to be carried out by a hybrid iterative PKS-NRPS, and a single set of the functional domains KS-AT-DH-KR-ACP that iteratively incorporate six malonyl-CoA to form two polyene chains, which were further condensed with the two free amine groups of L-ornithine *via* the NRPS activity. This resulted in a tetramate-polyene intermediate, which was then cyclized *via* reduction by the tailoring oxidative enzymes to form the PTM skeleton. The potential biosynthetic gene cluster and the homologs of the *A. hymeniacidonis* core PKS-NRPS protein (TL08_RS13440) identified in the NCBI database and their phylogenetic relationships are depicted in Supplementary Figure S1 and Supplementary Table S2, respectively.

### Fermentation, Isolation, and Characterization of Bioactive Tetramates From *Actinoalloteichus hymeniacidonis*

To test whether the tetramate gene cluster identified through genome mining is functional in *A. hymeniacidonis*, we conducted a small-scale fermentation (10 ml) and confirmed the production of possible PTMs supported by LC-MS analyses (Supplementary Figure S3). To characterize the active compounds, *A. hymeniacidonis* was cultivated in a 6 L scale to yield a crude extract subjected to separation by chromatography on silica gel and Sephadex LH-20 columns, yielding pure compound 1 (0.8 mg).

Compound 1 was isolated as a major component. HRESIMS data of 1 ([M + H]^+^ 511.2788, calculated for 511.2803, Δ 2.7 ppm) suggested a molecular formula of C_29_H_38_N_2_O_6_ and implied that it might be xanthobaccin A through AntiBase search (Laatsch, 2012). Four olefinic protons (H-8, δ 5.93; H-9, δ 5.74; H-23, δ 6.63; H-24, δ 6.97) corresponding to two double bonds were observed. A *trans* and a *cis* configuration for the two double bonds was deduced by the coupling constants (16.0 and 10.8 Hz between H23/H24 and H8/H9, respectively). In the HMBC spectrum, NH (δ 7.87, t, 5.6 Hz), H-8, and H-9 showed correlations to C-7 (δ 166.1). The ^13^C NMR spectrum indicated the presence of a tetramate unit by the presence of signals for C-1 (δ 196.2), C-27 (δ 178.5), C-2 (δ 61.4), and C-26 (δ 99.4). The location of a carbonyl group at C-20 (δ 207.9) was established by HMBC correlations between H-12, H-21 and H-22 and C-20. The aliphatic parts of the molecule were confirmed by both COSY and HMBC correlations. The selected HMBC correlations can be seen in Supplementary Figure S4. Both ^1^H NMR and ^13^C NMR spectra were identical to xanthobaccin A reported in the literature (Hashidoko et al., 1999). Xanthobaccin A was firstly reported from the Gram-negative bacterium *Stenotrophomonas* sp. SB-K88 living in the rhizosphere, which exhibited high activity against the plant fungal pathogen *Pythium ultimum* (Hashidoko et al., 1999). Here, for the first time, we demonstrated that xanthobaccin A is also produced by a Gram-positive bacterium associated with a sponge. Together with the discovery of the cytotoxic ikarugamycins and clifednamide A from a sponge-associated *Streptomyces* sp. (Dhaneesha et al., 2019), it indicates that sponge endosymbionts might be the true producers for those PTMs reported from sponges, such as the antinematode geodin A (Capon et al., 1999).

### Antimicrobial Mechanisms of Tetramates

Xanthobaccin A was reported to be the principal active metabolite of *A. hymeniacidonis*. The compound exhibited a minimum inhibitory concentration (MIC) of 1 μg/ml against *Pythium ultimum* (Hashidoko et al., 1999). To compare the antibacterial activity of xanthobaccin A to other related tetramates, equesetin and ikarugamycin, were tested against *Bacillus subtilis*. All three tetramates had antimicrobial activity against *B. subtilis* with MIC values of 10, 0.62, 2.5 μg/ml for xanthobaccin A, equestin and ikarugamycin, respectively.

Next, we tested the ion-chelating activity of all three tetramates by adding ferric citrate to the solutions of tetramates, followed by HPLC analyses. However, it was hard to observe the corresponding stoichiometry under acidic conditions. Thus, commercially available tetramates equisetin and ikarugamycin were further analyzed for their iron complex under a neutral HPLC condition.

**FIGURE 3:**
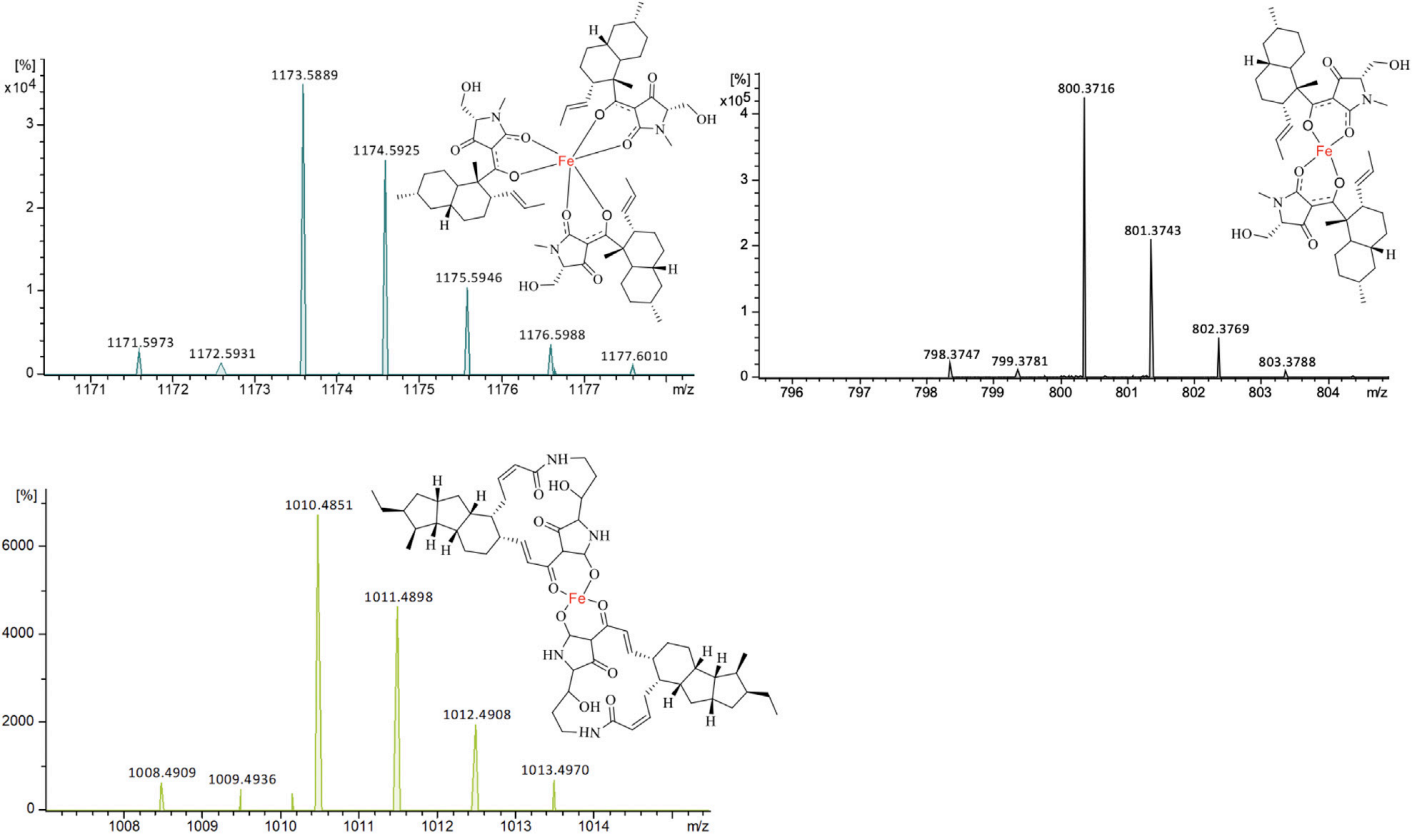
ESIMS for the two different stoichiometries of equisetin-Fe(III) complexes and one ikarugamycin-Fe(III) complex. **(A)** [M + H]^+^, m/z 1,171.5993, Fe(III)(equisetin)_3_; **(B)** [M + H]^+^, m/z 798.3747, Fe(III)(equisetin)_2._ **(C)** [M + H]^+^, m/z 1,008.4909, Fe(III)(ikarugamycin)_2_.

Through extracted ion chromatography, both Fe(III)(equisetin)_3_ (m/z 1,171.5973 [M + H]^+^, calc. 1,171.5993 for C_66_H_90_N_3_O_12_Fe, Δ 1.7 ppm) and Fe(III)(equisetin)_2_ (m/z 798.3720 [M + H]^+^, calc. C_44_H_59_N_2_O_8_Fe 798.374, Δ 2.6 ppm) could be observed (Figure 3). It is not surprising since there are two different enolic tautomers of equisetin which lead to two or three complex structures. A higher electron density on the amide carbonyl compared to the carbonyl on the C-4 position could lead to the observation of a dominant stoichiometry under a neutral pH condition.

Different from equisetin, the relative larger tetramate ikarugamycin could form Fe(III)(ikarugamycin)_2_ (m/z 1,008.4909 [M + H]^+^, calc. C_58_H_73_FeN_4_O_8_ 1,008.4897, Δ 1.2 ppm) as the dominant iron complex (Figure 3). During the submission of the manuscript, another study (Yu et al., 2021) reported that HSAF could act as an iron-chelator with a similar chelating pattern. Also, the HSAF-mutant was less susceptible to oxidative stress (Yu et al., 2021).

We hypothesized that there is a similar scenario compared to the copper bis(tenuzonate) structure. Revisiting the former investigation of tenuazonic acid-Fe (III) complex revealed a similar observation, where ions derived from a loss of fragment radicals were detected (Lebrun et al., 1985). In the same investigation, a reduction process of Fe(III)(TA)3 to Fe(II)(TA)3 was proposed in the report (Lebrun et al., 1985). This led us to our hypothesis that the Fenton chemistry follows the complexation of Fe(III) with tetramates.

To further address the function of tetramates, we studied the previous evidence from HSAF in *Candida albicans* IBT656, where a transcriptomics analysis was carried out. RNA-seq of PTM-treated *Candida albicans* revealed that HSAF triggered apoptosis *via* ROS-dependent pathway (Ding et al., 2016). However, the exact mechanism remains unknown.

Nevertheless, this evidence and the iron-chelating activity pointed to a possible link to Fenton chemistry, which describes the oxidative degradation of organic matter by hydrogen peroxide (H_2_O_2_) in the role of Fe^2+^ under acidic conditions, first discovered by H. J. Fenton in 1894. H_2_O_2_ is naturally produced by living organisms, from bacteria, algae to human phagocytic cells. Although H_2_O_2_ has limited reactivity, in the presence of Fe^2+^, it can initiate a very strong reaction to produce high active hydroxyl radicals.

**FIGURE 4:**
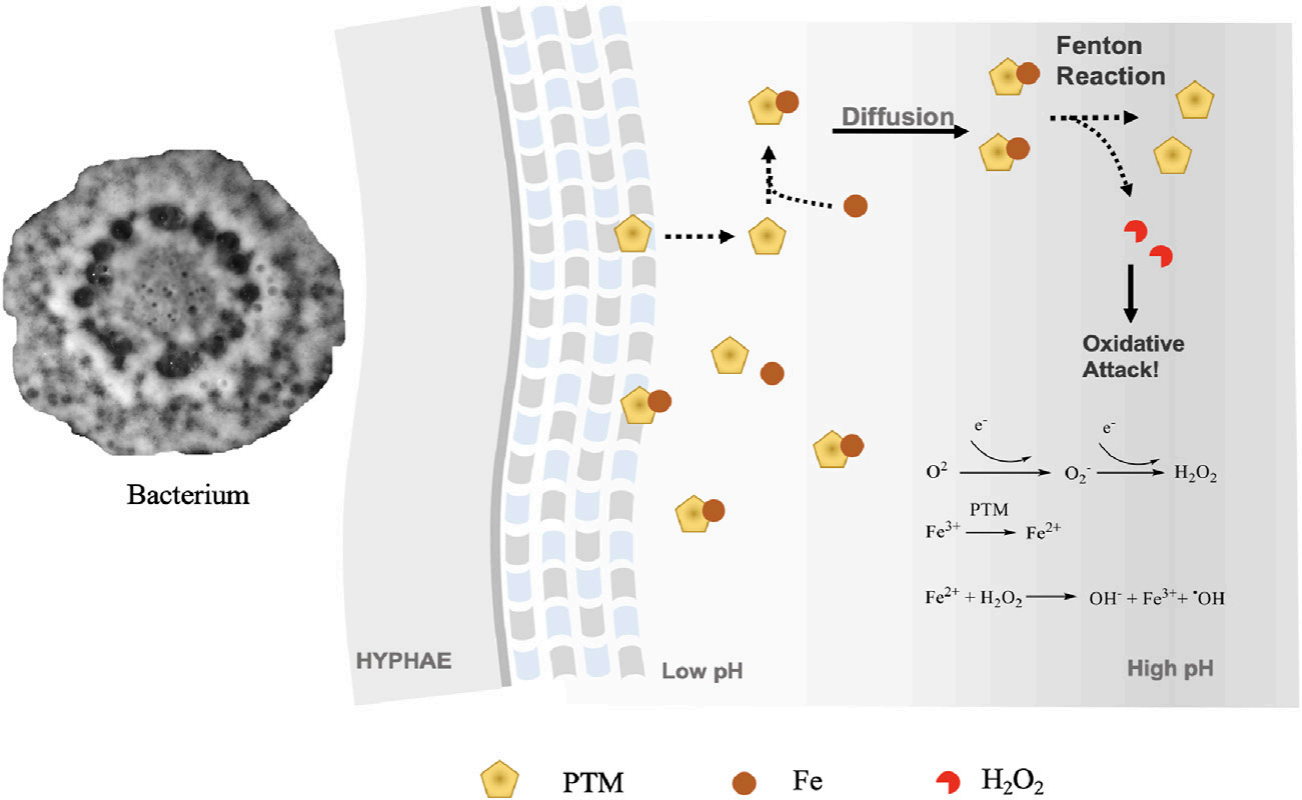
Proposed mode-of-action of PTMs as metallophores and antibiotics. Bacteria secrete PTMs into extracellular environment. As the diffusion occurs and the pH lowers, PTMs start to reduce Fe^3+^ and initiate a Fenton’s reaction. An oxidative attack could occur, where PTMs function as antibiotics.

To clarify the iron-reducing activities, we carried out an iron-reducing experiment with ferrozine. As ferrozine forms a pink complex with iron II, which can be analyzed by a UV spectrometer, we determined the iron III reducing activity of the three tetramates using the ferrozine method. All tetramates showed positive results in the ferrozine test. Upon incubation of xanthobaccin A with ammonium iron III citrate and ferrozine, a ferrozine-iron II complex was detected by HPLC-HRMS, which showed a characteristic UV maximum absorption at 562 nm and formula of C_40_H_28_FeN_8_O_12_S_4_ ([M + H]^+^ m/z 995.0203, Δ-2.5ppm) (Supplementary Figure S5). This suggested the reduction of iron III to iron II by xanthobaccin A. It is likely that the complexation of xanthobaccin with iron II triggers Fenton chemistry (Figure 4) and produces reactive hydroxyl radicals as depicted in Figure 4. As expected, adding the radical scavenger vitamin C reduced the antibacterial effects of tetramates. After adding vitamin C, the inhibition zones were reduced from 23 to 18 mm for equisetin, and 7 to 6 mm for xanthobaccin A, respectively.

## Discussion

Many natural metallophores play important roles as virulence factors, signaling molecules, and regulators of oxidative stress (Johnstone and Nolan 2015). Among those metallophores with potent antimicrobial activities, tetramates could be novel potential antibiotics. Tetramic acids possessing a 3-acyl group are supposed to chelate trivalent metal ions (Markopoulos et al., 2010; Zaghouani and Nay 2016). A previous report demonstrated that anti-HIV (Miller et al., 1963), antibacterial, and antitumoral agent tenuazonic acid (Gitterman 1965) isolated from *Alternaria tenuis* could form copper salts (Rosett et al., 2015). Similarly, the antibacterial agent magnesidin from *Pseudomonas magnesiorubra* nov sp. could form metal complexes with Mg, Cu, Ni, and Fe (Kohl et al., 1974).

PTMs are an important emerging family of bioactive compounds described solely in bacteria. Their potential as an iron-chelator has, to our knowledge, not been investigated before, and we here, for the first time, demonstrated that PTMs are a group of bacterial metallophores. By an iron-reducing experiment with ferrozine, the data support that xanthobaccin A, equisestin, and ikarugamycin can induce Fenton chemistry, which could be alleviated by adding the radical scavenger vitamin C. The Fenton reaction *in vivo* appears to occur in the presence of catalytic ferrous iron, leading to the production of the most reactive hydroxyl radicals in the biological system (Dizdaroglu 1991). The hydroxyl radical has a very short *in vivo* half-life of approximately 10^–9^ s and high reactivity (Sies 1993). It can damage virtually all types of macromolecules including carbohydrates, nucleic acids, lipids, and amino acids (Reiter et al., 1995). This makes it the most harmful free radical for the organism (Reiter et al., 1997).

Interestingly, microorganisms also use Fenton chemistry for defense, and besides tetramates, there are other examples. Co-cultivation of the model saprotrophic basidiomycete *Serpula lacrymans* with a ubiquitous terrestrial bacterium, either *Bacillus subtilis*, *Pseudomonas putida*, or *Streptomyces iranensis* could induce the production of the antibacterial compound atromentin (Tauber et al., 2016), a group of pigments that could trigger Fenton chemistry (Shah et al., 2015).

Since PTMs can be detected in both sugar beet rhizosphere soil and sponges (Nakayama, 1996; Capon et al., 1999), we hypothesize that higher organisms, can recruit bacterial PTM producers for a chemo-defense against other organisms. Given the metallophore and antibiotics activity, we propose that one potential ecological role of PTMs in the natural ecosystem is to chelate Fe^3+^ in the vicinity of hyphae at low pH, which restrains the reduction of Fe^3+^ and initiation of Fenton chemistry on-site (Figure 4). The piracy of neighboring Fe^3+^ causes the limitation of competing organisms. A decreasing gradient of tetramates concentration by diffusion away from the hyphae with subsequent increase in pH will result in the dissociation of Fe^3+^-tetramate chelates, thus initiating a Fenton reaction for an oxidative attack. Given the wide existence and effective functions of Fenton chemistry in the ecosystem, tetramates producers might be developed as solutions for biocontrol against crop infections.

## Conclusion

We isolated an antimicrobial agent xanthobaccin A from a sponge endosymbiont. For the first time, we demonstrated that bacterial PTMs can function as metallophores. Xanthobaccin A, equestin, and ikarugamycin exhibited antibacterial activity against *B. subtilis* and the effects could be alleviated by adding radical scavenger vitamin C. We demonstrated that all three tetramates could trigger Fenton chemistry, and this potentially explains why tetramates display broad biological activity. The isolation of PTMs from a sponge bacterial endophyte provides indirect evidence that the sponge-associated bacteria could be the true producers of sponge-derived PTMs. We propose that tetramates may function as a natural defense of niches by growth inhibition of other microbes via Fenton Chemistry. They could be potentially developed as effective antibiotics against drug-resistant pathogens (Samuni et al., 1983).

## Data Availability

The original contributions presented in the study are included in the article/Supplementary Material, further inquiries can be directed to the corresponding author.
