# Supplementary material for: Polycyclic Tetramate Macrolactams—A Group of Natural Bioactive Metallophores
Source: Front Chem. 2021 Nov 12;9:772858. doi: 10.3389/fchem.2021.772858 (PMC8632820; doi:10.3389/fchem.2021.772858)
Supplement: Supplementary file 1 [file DataSheet1.pdf]

# Supplementary materials

## Polycyclic tetramate macrolactams–a group of natural bioactive metallophores

**Ling Ding[1,*], Sheng-Da Zhang[1], Ahmad Kasem Haidar[1], Manila Bajimaya[1], Yaojie Guo[1], Thomas Ostenfeld Larsen[1] and Lone Gram[1]**

[a] Department of Biotechnology and Biomedicine, Technical University of Denmark, Søltofts Plads bldg. 221, DK-2800 Kgs Lyngby, Denmark

*** Correspondence:**
Corresponding Author
lidi@dtu.dk

**Table. S1**. Individual genes from the proposed PTM gene cluster in *Actinoalloteichus hymeniacidonis*.

| Protein ID | Proposed function |
|---|---|
| WP_069849309.1 | *MarR_family transcriptional_regulator* |
| WP_069849311.1 | *prolyl aminopeptidase* |
| WP_069853659.1 | *hypothetical protein* |
| WP_084642982.1 | *cellulose-binding protein* |
| WP_069849315.1 | *DUF664 domain-containing protein* |
| WP_069853660.1 | *Hydrolase* |
| WP_069849318.1 | *hypothetical protein* |
| WP_069849320.1 | *hypothetical protein* |
| WP_084642983.1 | *sterol desaturase family protein* |
| WP_069849322.1 | *t1pks-nrps* |
| WP_069853662.1 | *"NAD(P)/FAD-dependent oxidoreductase* |
| WP_084642984.1 | *NAD(P)/FAD-dependent oxidoreductase* |
| WP_069849326.1 | *crotonyl-CoA_reductase_/_alcohol_dehydrogenase* |
| WP_069849328.1 | *siderophore-interacting protein* |
| WP_069849330.1 | *iron-siderophore ABC transporter substrate-binding protein* |
| WP_069849332.1 | *hypothetical protein* |
| WP_069849334.1 | *YeeE/YedE family protein* |
| WP_069849336.1 | *hypothetical protein* |
| WP_084643660.1 | *MFS transporter* |
| WP_069849338.1 | *methionine ABC transporter substrate-binding protein* |
| WP_069853663.1 | *methionine ABC transporter ATP-binding protein* |
| WP_069849340.1 | *methionine ABC transporter ATP-binding protein* |

**Table. S2**. Selected proteins of the core PKS-NRPSs from different bacteria for construction of the PKS-NRPS phylogenetic tree. EntF from rapamycin-biosynthesis gene cluster was used as an outsource.

| Accession number | Organism |
|---|---|
| WP_099405199.1 | *Chitinimonas* sp. |
| WP_069849322.1 | *Actinoalloteichus hymeniacidonis* |
| WP_011470195.1 | *Saccharophagus degradans* |
| WP_051746124.1 | *Streptomyces scopuliridis* |
| WP_116023996.1 | *Actinomadura umbrina* |
| WP_114248145.1 | *Streptomyces atratus* |
| WP_072486961.1 | *Streptomyces atratus* |
| WP_032756822.1 | *Streptomyces alboviridis* |
| WP_015612794.1 | *Streptomyces fulvissimus* |
| WP_030638588.1 | *Streptomyces flavovirens* |
| AJD77023.1 (*IkaA*) | *Streptomyces* sp. ZJ306 |
| APT37060.1 (*IkaA*) | *Streptomyces* sp. |
| WP_078635587.1 | *Streptomyces* sp. NRRL F-2890 |
| WP_078847110.1 | *Streptomyces xiamenensis* |
| WP_100586210.1 | *Kitasatospora* sp. CB02891 |
| WP_043910558.1 | *Kitasatospora griseola* |
| WP_050361965.1 | *Streptomyces europaeiscabiei* |
| WP_030572898.1 | *Streptomyces cyaneofuscatus* |
| WP_030226717.1 | *Actinoalloteichus cyanogriseus* |
| WP_128845715.1 | *S. lavendulae* |
| WP_093843627.1 | *Streptomyces harbinensis* |
| WP_074474637.1 | *Micromonospora carbonacea* |
| WP_123603417.1 | *Micromonospora* sp. |
| WP_093154928.1 | *Saccharopolyspora antimicrobica* |
| WP_010311945.1 | *Saccharopolyspora spinosa* |
| WP_077004069.1 | *Saccharothrix* sp. |
| WP_093355332.1 | *Saccharothrix* sp. |
| WP_033434304.1 | *Saccharothrix syringae* |
| WP_035282712.1 | *Actinokineospora spheciospongiae* |
| WP_110079926.1 | *Actinokineospora mzabensis* |
| WP_086782311.1 | *Crossiella equi* |
| WP_098511016.1 | *Amycolatopsis sulphurea* |
| WP_120025505.1 | *Amycolatopsis* sp. |
| WP_093942096. | *Actinoalloteichus hoggarensis* |
| WP_082124235.1 | *Lysobacter capsici* |
| WP_082648427.1 | *Lysobacter capsici* |
| WP_082723461.1 | *Lysobacter capsici* |
| WP_082578124.1 | *Lysobacter* sp. Root690 |
| WP_083512548.1 | *Lysobacter gummosus* |
| WP_079248388.1 | *Lysobacter antibioticus* |
| WP_123647640.1 | *Lysobacter enzymogenes* |
| WP_082644542.1 | *Lysobacter enzymogenes* |
| WP_121823893.1 | *S. rapamycinicus* |

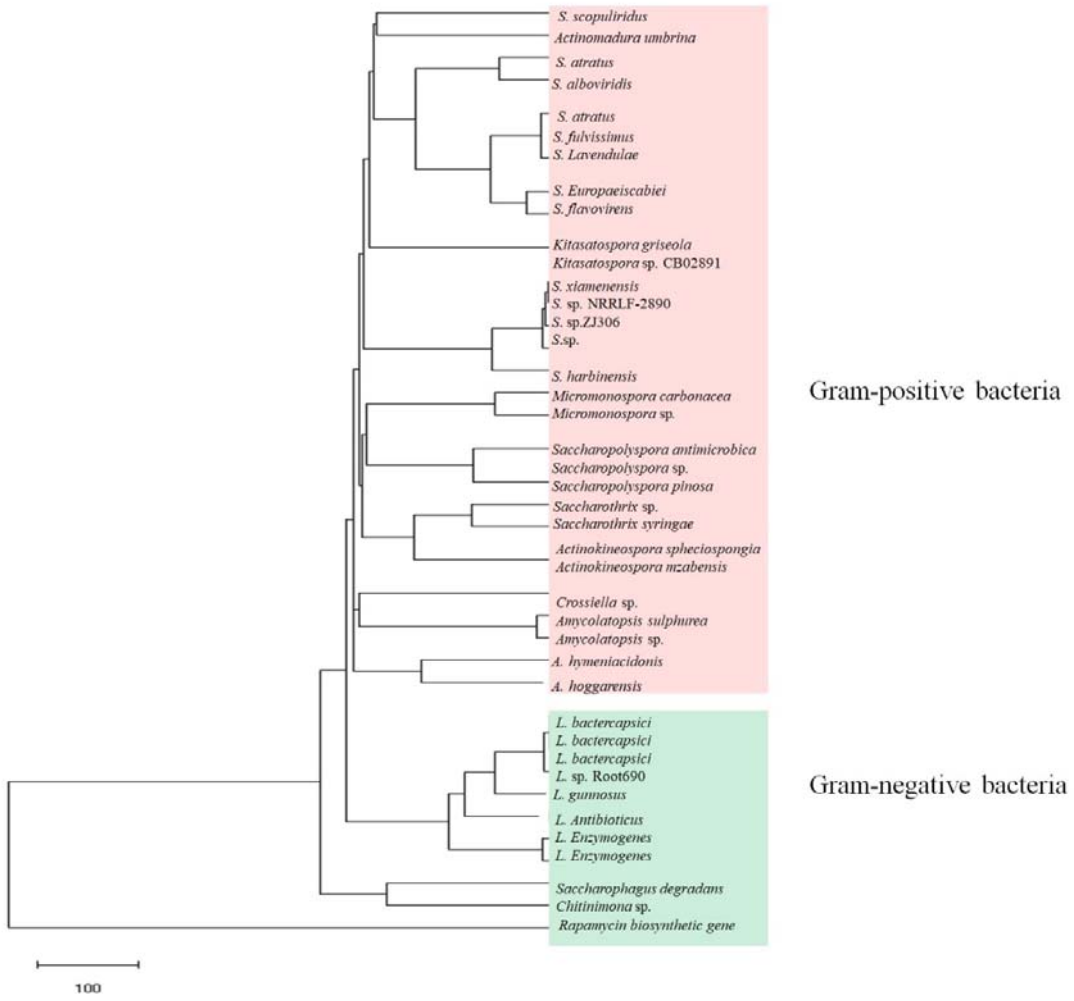


**Fig. S1.** Phylogenetic tree of the key PKS-NRPS genes selected from both Gram positive and Gram negative bacteria. The evolutionary history was inferred using the Neighbor-Joining method.[3] The optimal tree with the sum of branch length = 1250.37890625 is shown. The tree is drawn to scale, with branch lengths in the same units as those of the evolutionary distances used to infer the phylogenetic tree. The evolutionary distances were computed using the number of differences method[4] and are in the units of the number of base differences per sequence. The analysis involved 19 nucleotide sequences. Codon positions included were 1st+2nd+3rd+Noncoding. All positions containing gaps and missing data were eliminated. There were a total of 330 positions in the final dataset. Evolutionary analyses were conducted in MEGA X[3].

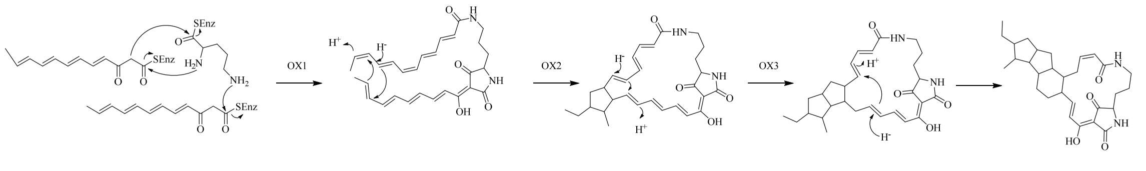


**Fig. S2.** Proposed Biosynthetic pathway for the xanthobaccin precursor (OX1-3: oxygenases).

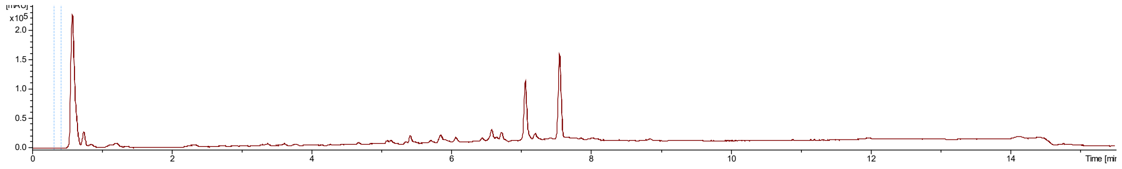

**Fig. S3.** HPLC profile of the ethyl acetate extract of *A. hymeniacidonis* showing the production of PTMs (wavelength for detection 324 nm, RT at 7.1 and 7.6 min, t7.6min xanthobaccin A; t7.1 min with formula of $C_{29}H_{38}N_2O_6$, a not fully identified PTM due to the low amount)

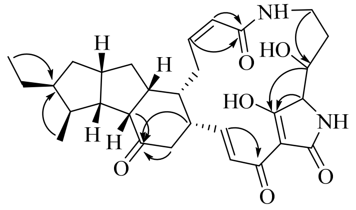


**Fig. S4.** Selected HMBC correlations for xanthobaccin A.

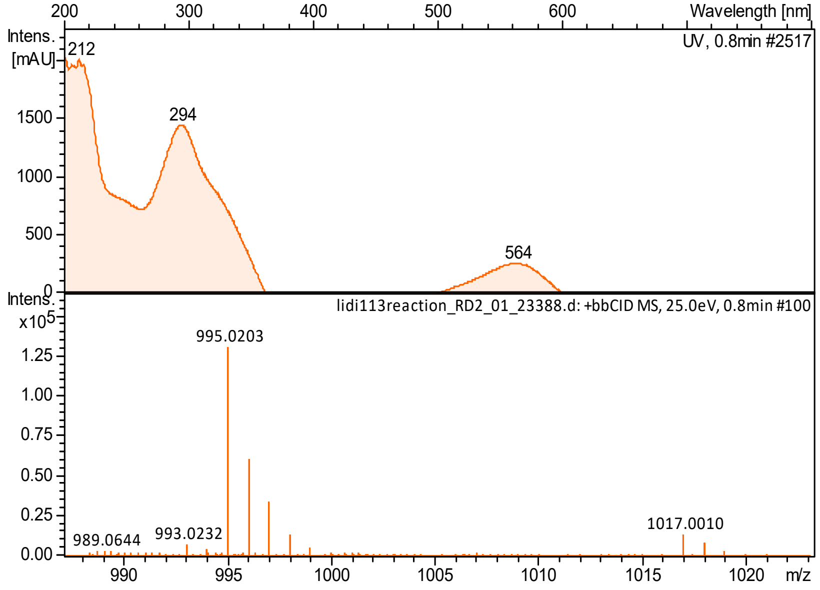


**Fig. S5.** Tetramates chelates and reduces iron. Top: characteristic UV absorption for ferrozine-ferrous complex after iron-reduction of xanthobaccin A; Below: the high-resolution mass spectrometry profile of ferrozine-ferrous.

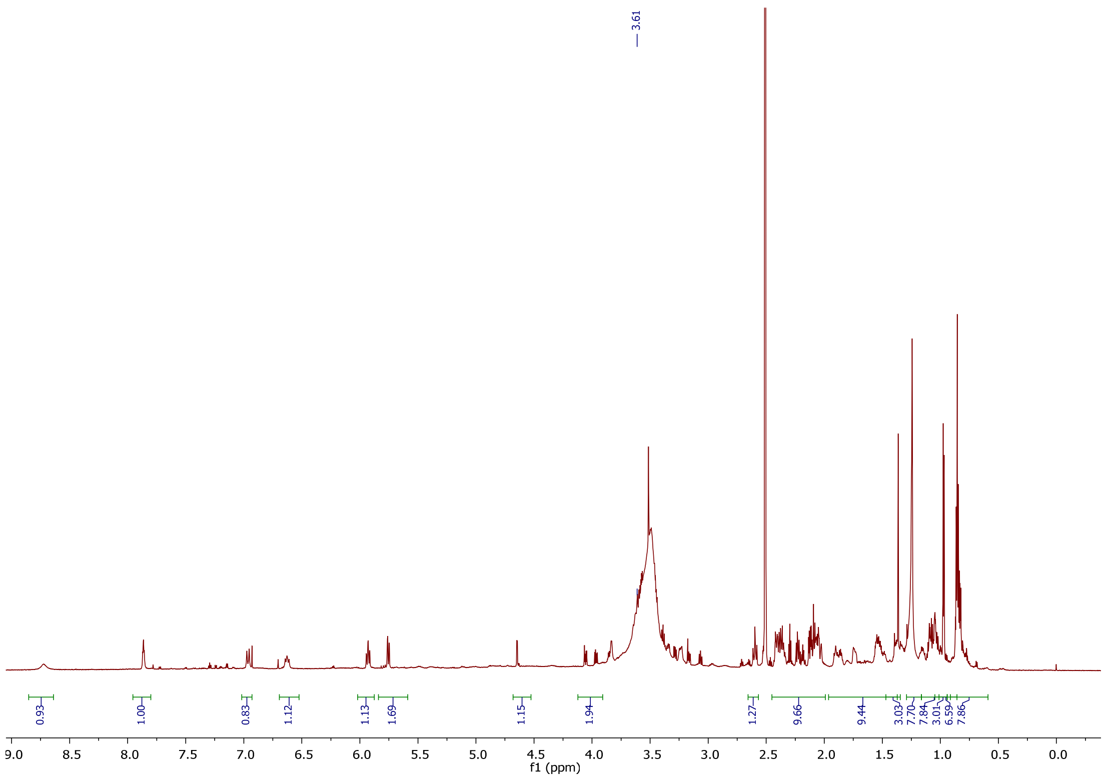


**Fig. S6a**. $^{1}$HNMR spectrum of xanthobaccin A.

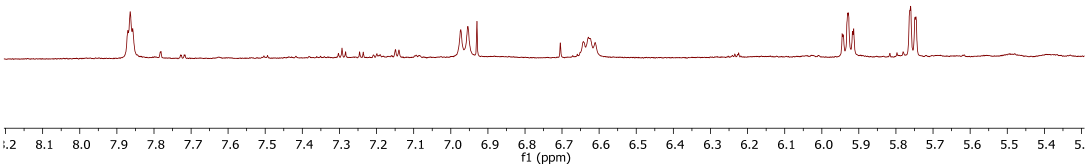


**Fig. S6b**. $^{1}$HNMR spectrum of xanthobaccin A.

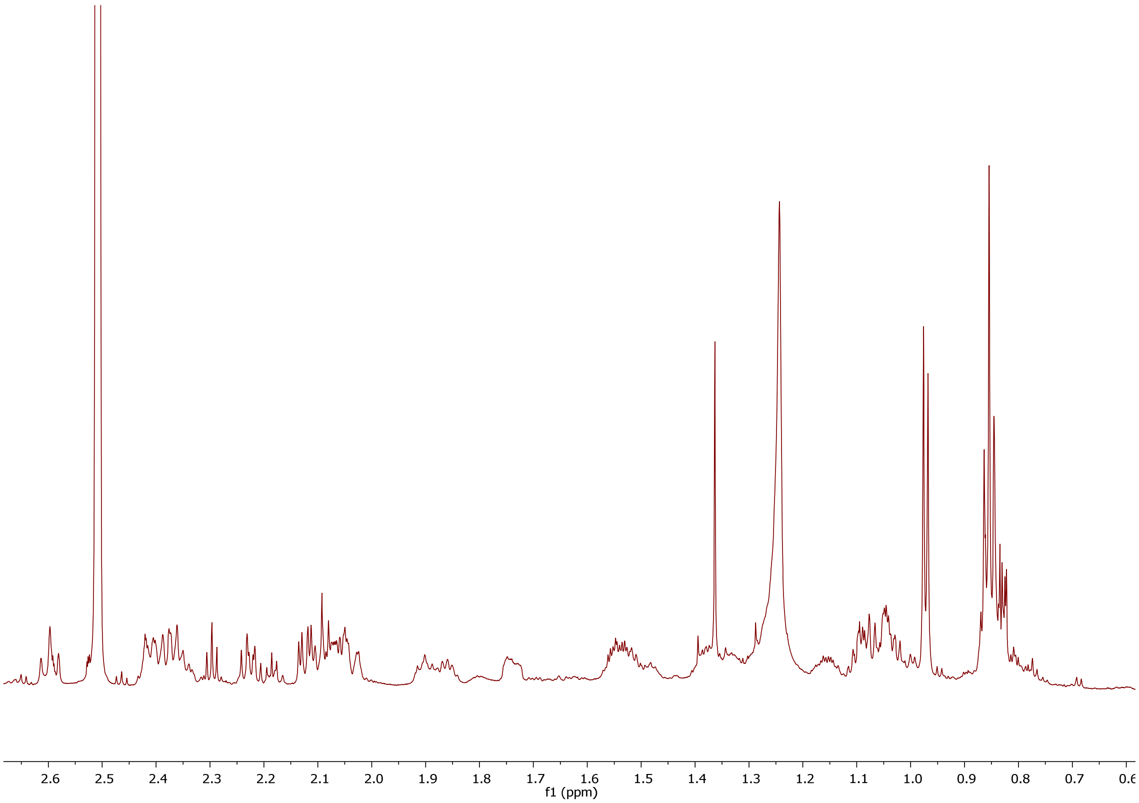


**Fig. S6c**. $^{1}$HNMR spectrum of xanthobaccin A.

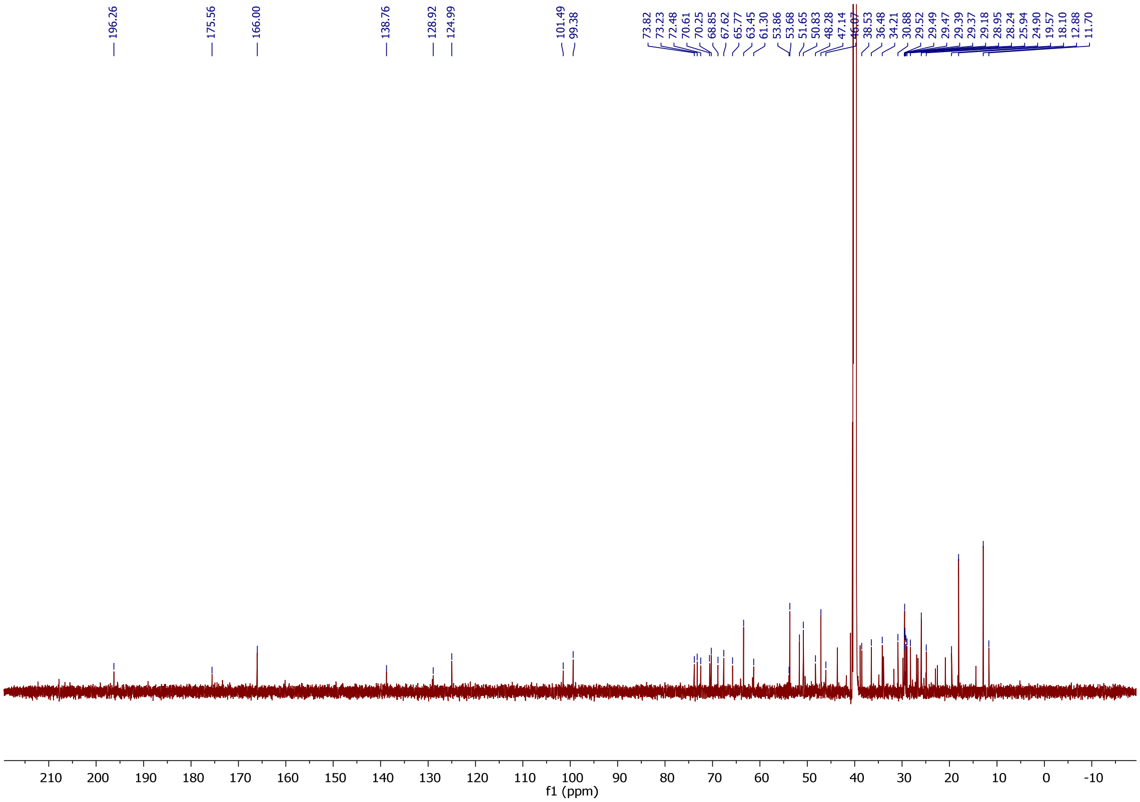


**Fig. S7**. $^{13}$CNMR spectrum of xanthobaccin A.

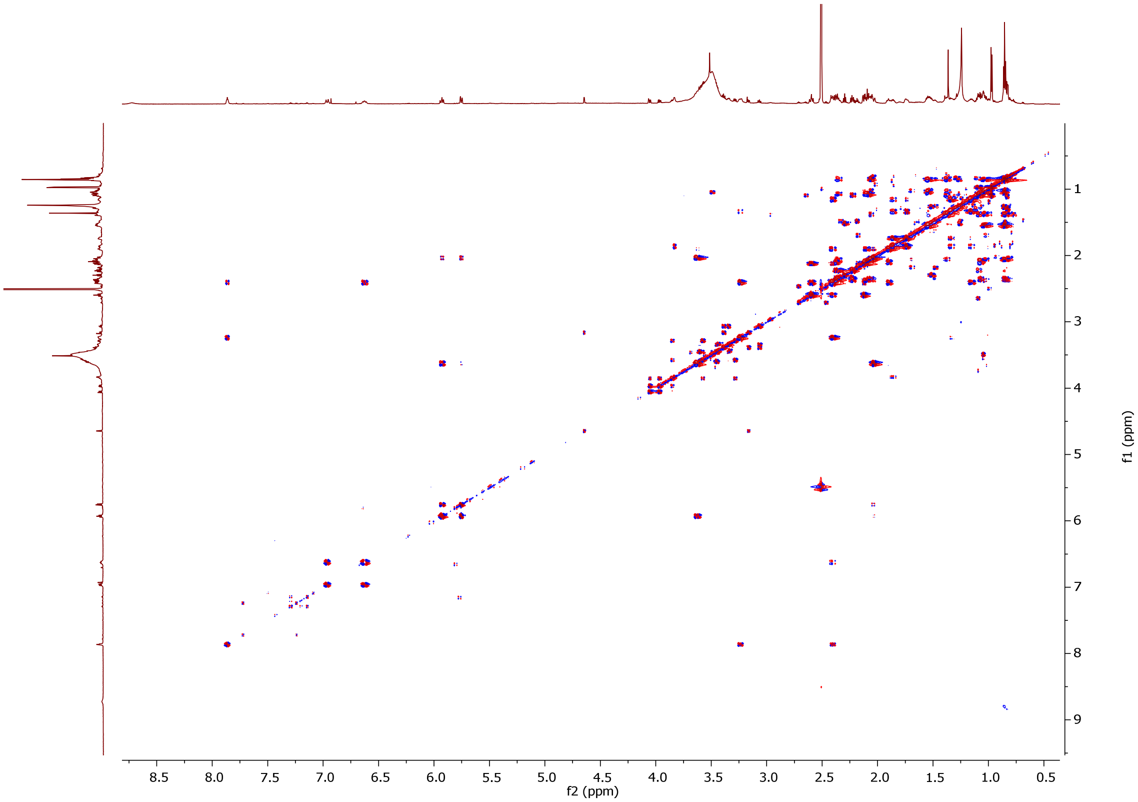


**Fig. S8a**. COSY spectrum of xanthobaccin A.

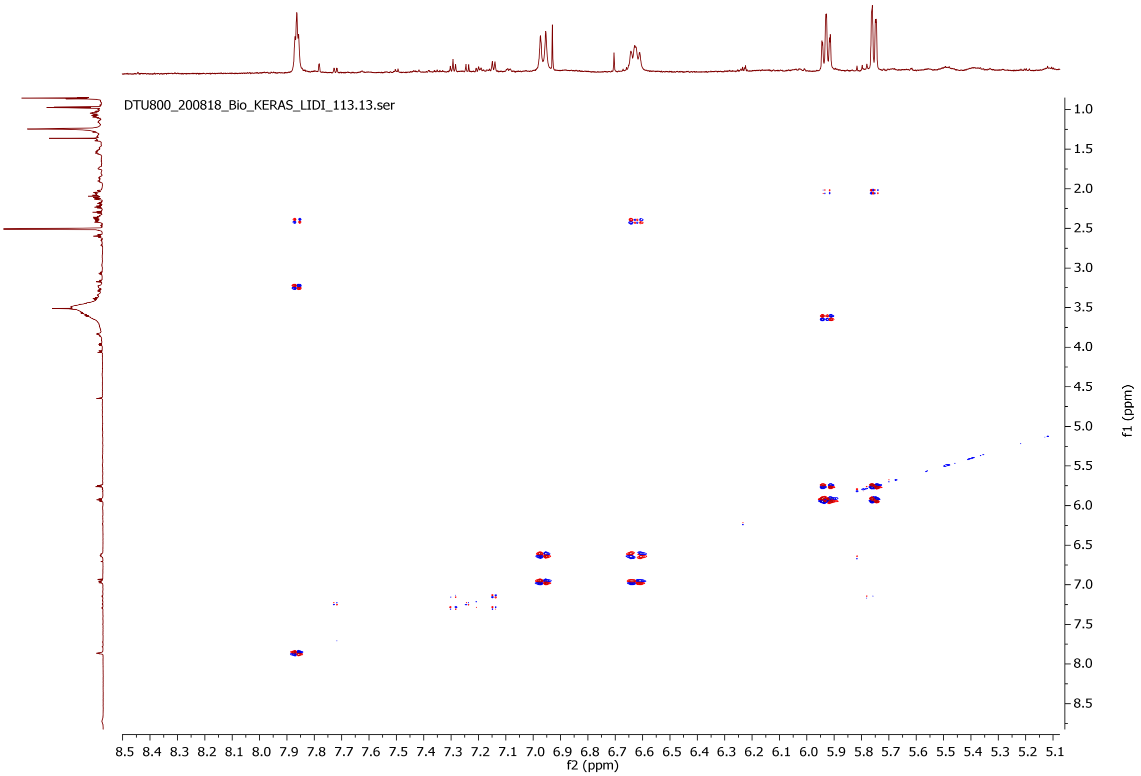


**Fig. S8b**. COSY spectrum of xanthobaccin A.

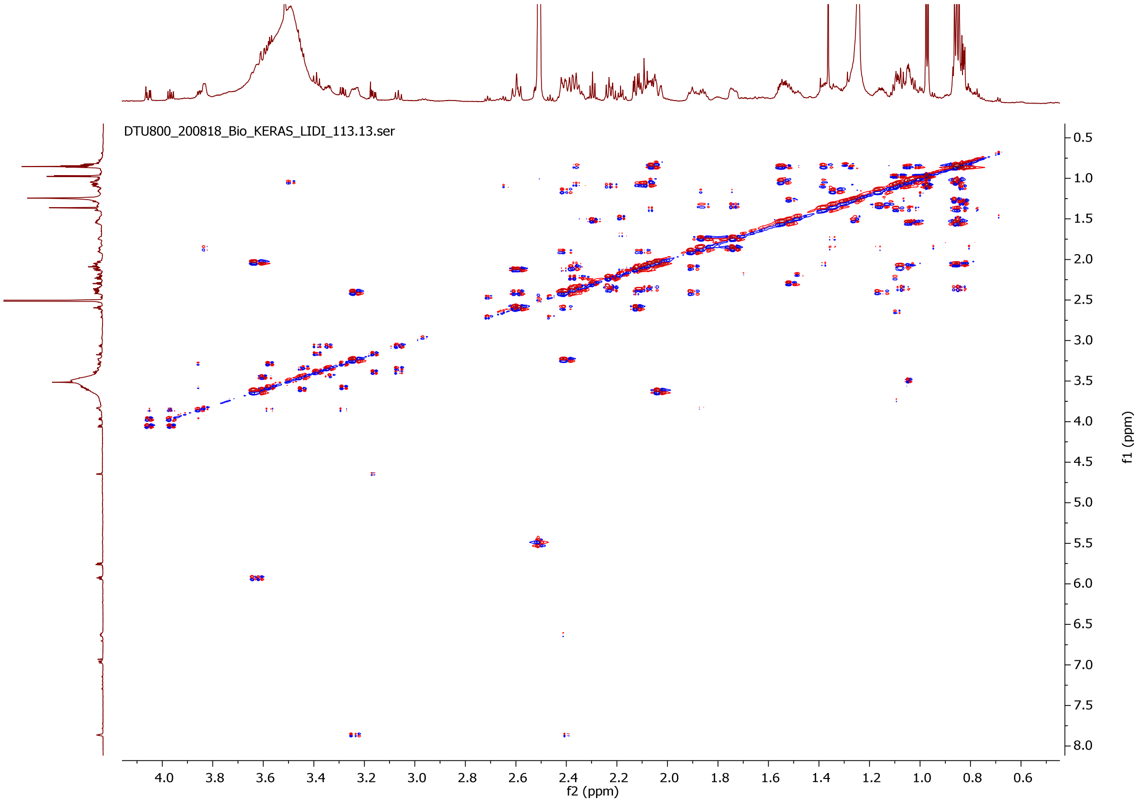


**Fig. S8c**. COSY spectrum of xanthobaccin A.

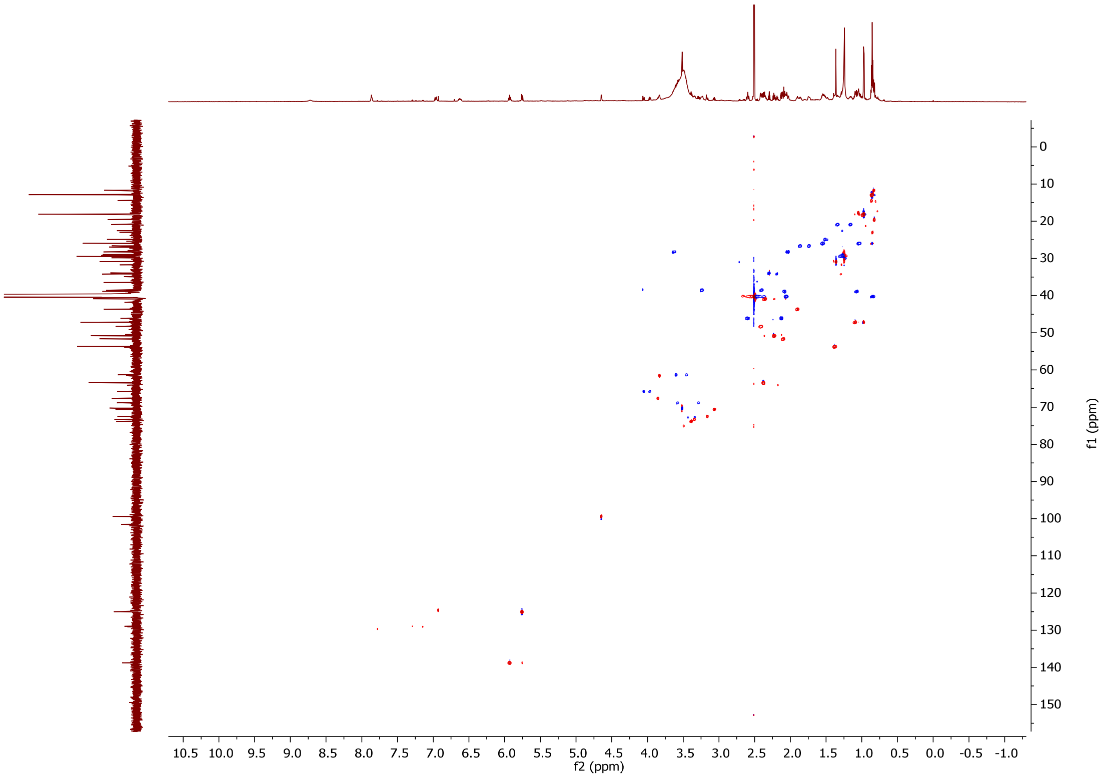


**Fig. S9a**. HSQC spectrum of xanthobaccin A.

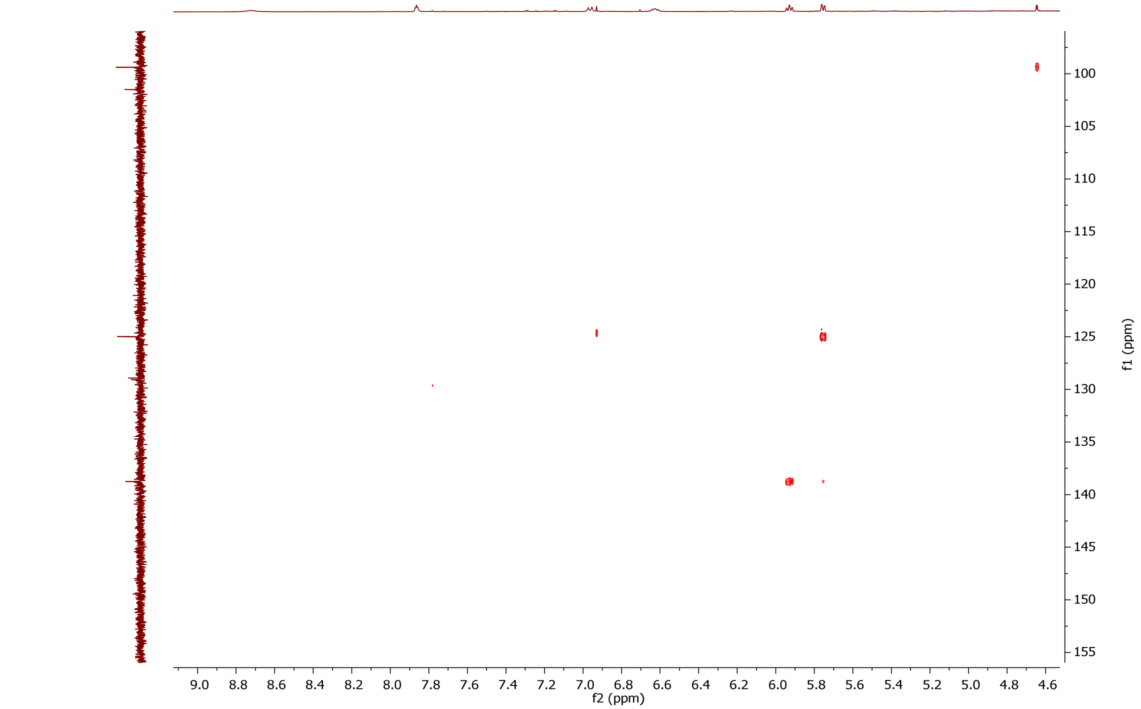


**Fig. S9b**. HSQC spectrum of xanthobaccin A.

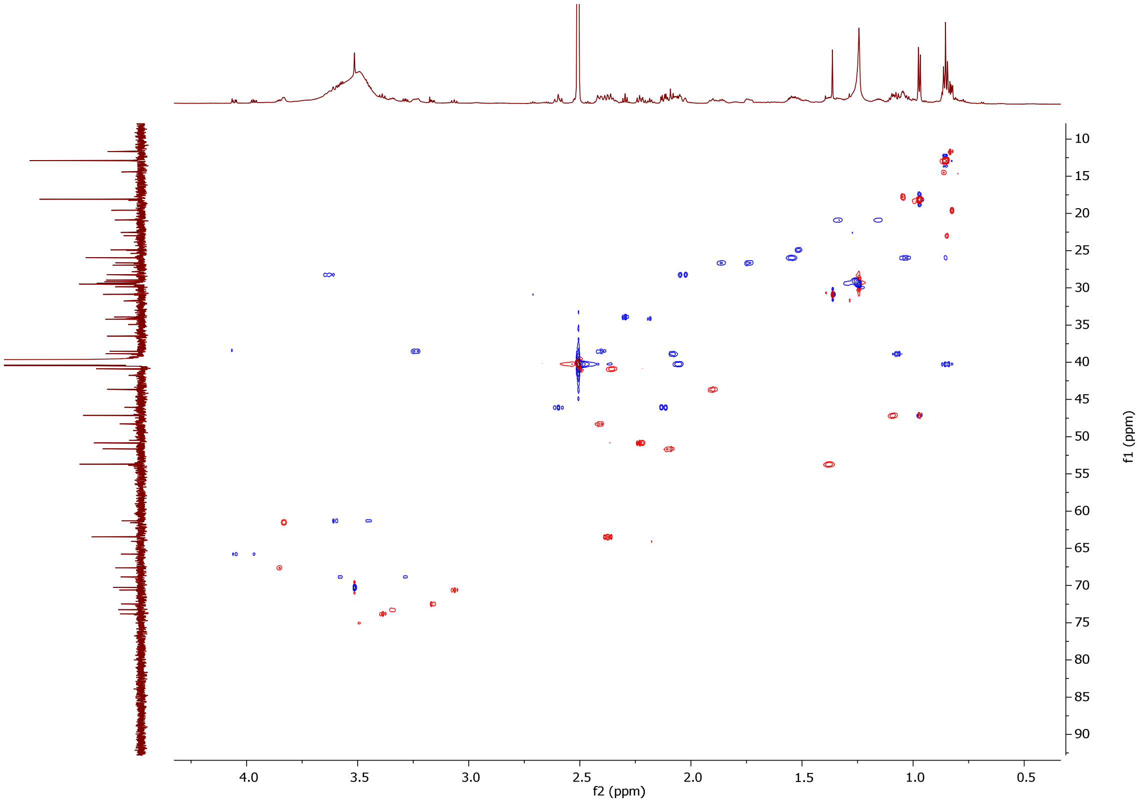


**Fig. S9c**. HSQC spectrum of xanthobaccin A.

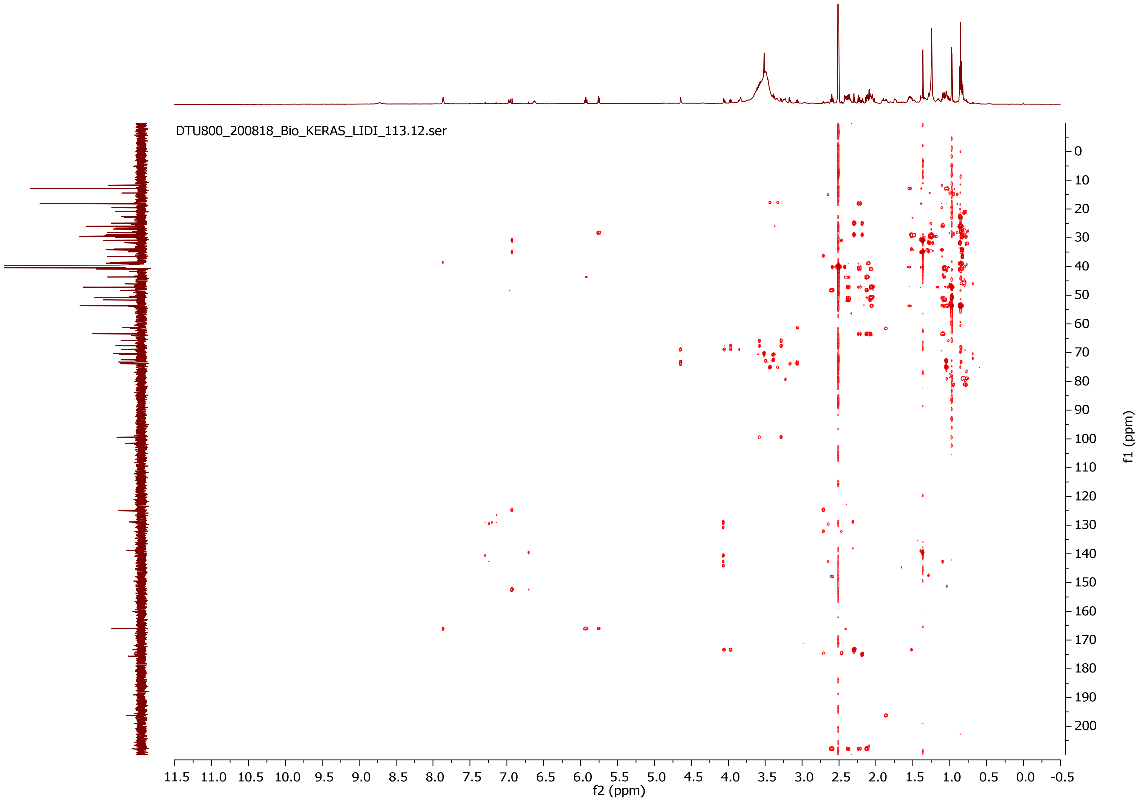


**Fig. S10a**. HMBC spectrum of xanthobaccin A.

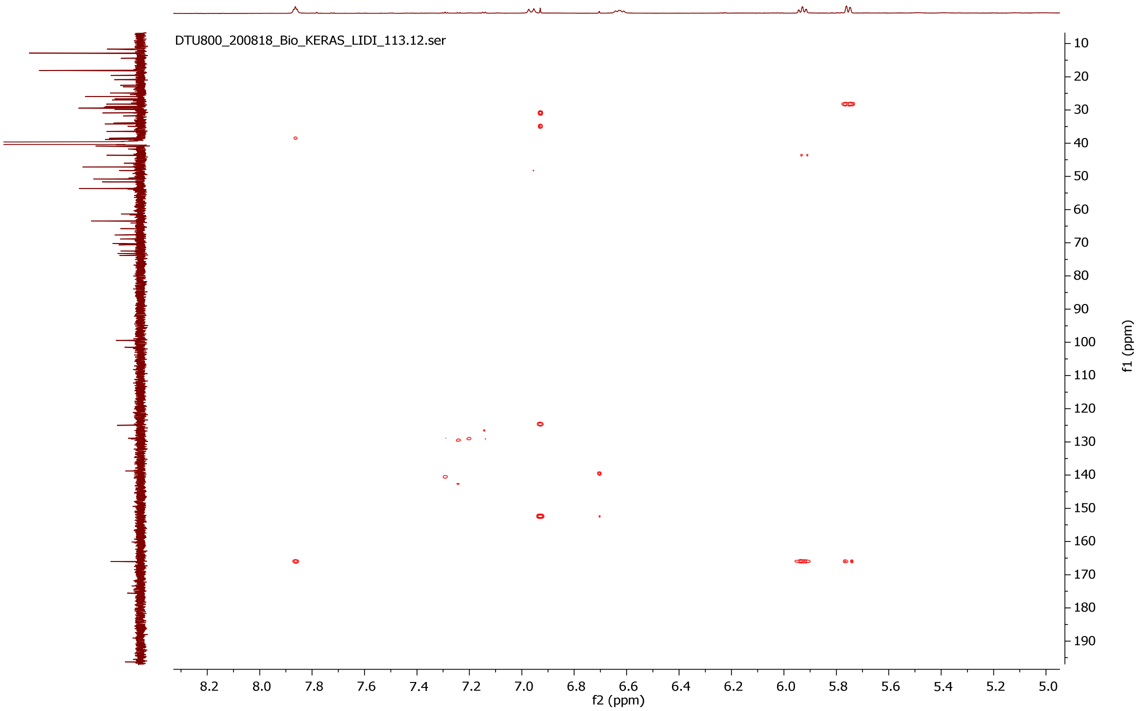


**Fig. S10b**. HMBC spectrum of xanthobaccin A.

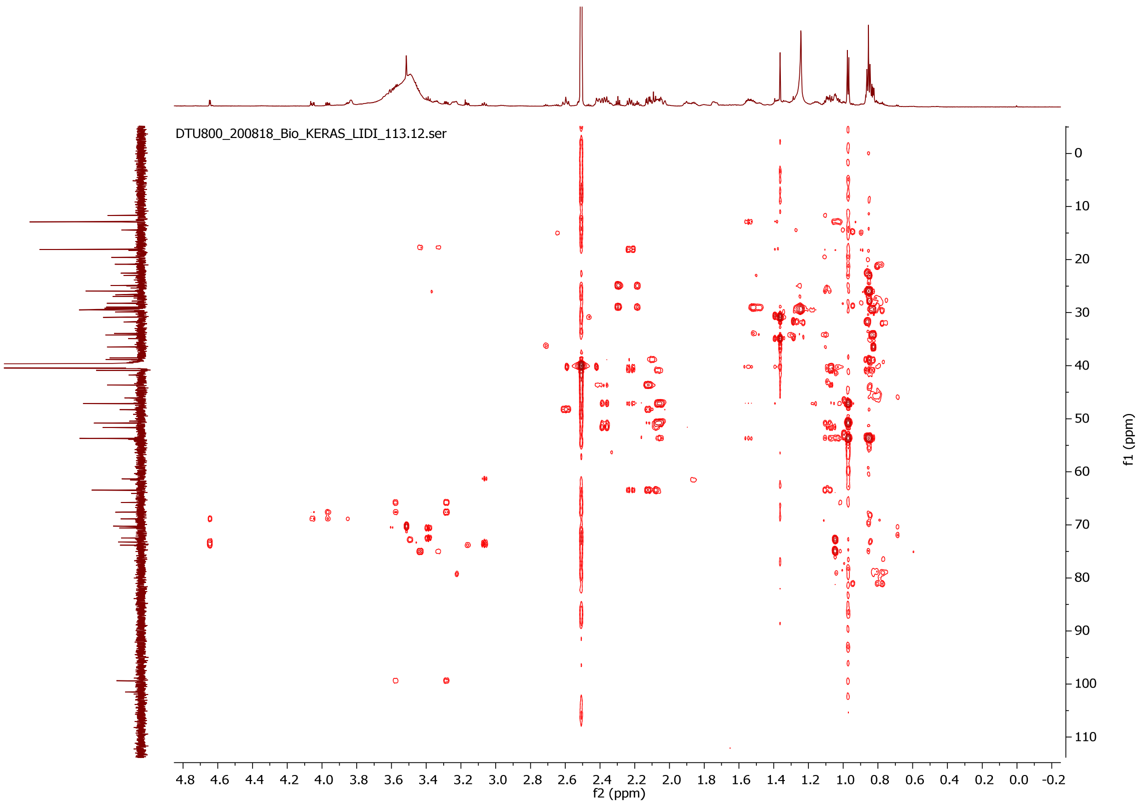


**Fig. S10c**. HMBC spectrum of xanthobaccin A.

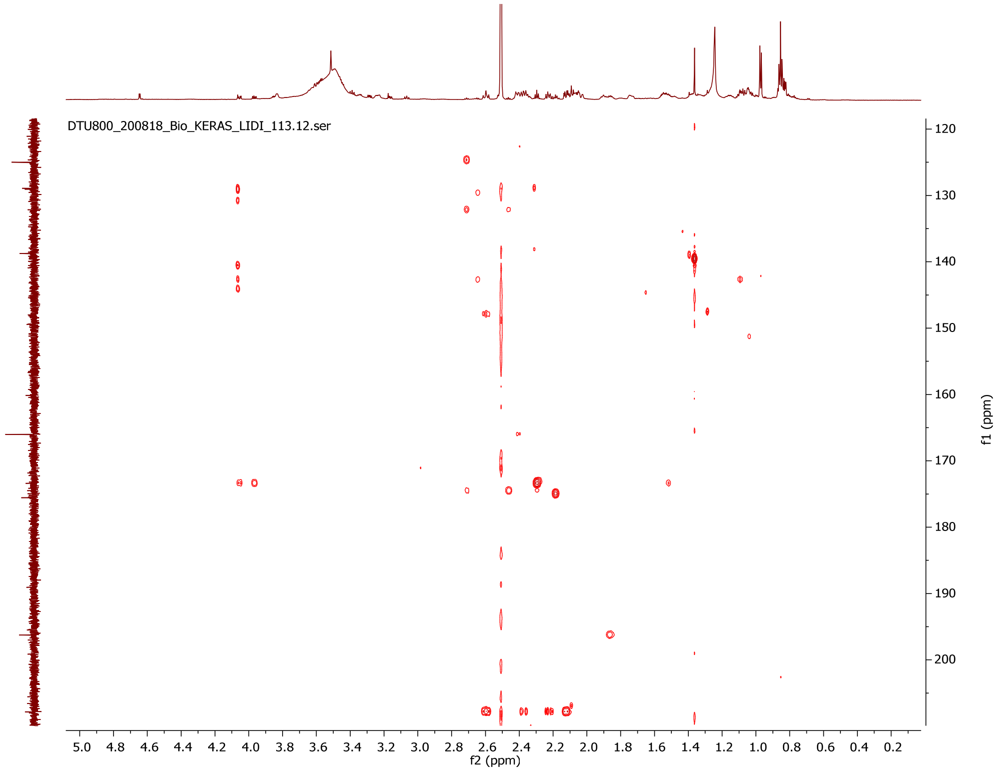


**Fig. S10d**. HMBC spectrum of xanthobaccin A.

## References


1. Saitou, N. Nei, M. (1987). doi:10.1093/oxfordjournals.molbev.a040454
2. Nei, M. Kumar S. *Mol. Evol. Phylogenetics. Oxford Univ. Press. New York.* (2000).
3. Kumar, S., Stecher, G., Li, M., Knyaz, C. Tamura, K. MEGA X: Molecular evolutionary genetics analysis across computing platforms. *Mol. Biol. Evol.* (2018). doi:10.1093/molbev/msy096
4. Kelley, L. A., Mezulis, S., Yates, C. M., Wass, M. N. Sternberg, M. J. E. The Phyre2 web portal for protein modeling, prediction and analysis. *Nat. Protoc.* (2015). doi:10.1038/nprot.2015.053
